# Evolutionary dynamics of the H7N9 avian influenza virus based on large-scale sequence analysis

**DOI:** 10.1371/journal.pone.0220249

**Published:** 2019-08-12

**Authors:** Jiasheng Xiong, Ping Zhao, Pengfei Yang, Qingli Yan, Lufang Jiang

**Affiliations:** 1 College of Marine Science, Shandong University (Weihai), Weihai, People’s Republic of China; 2 Department of Epidemiology, School of Public Health, Fudan University, Shanghai, People’s Republic of China; 3 Key Laboratory of Public Health Safety, Ministry of Education, Shanghai, People’s Republic of China; 4 Huai’an Center for Disease Control and Prevention, Huai’an, People’s Republic of China; Universiti Putra Malaysia, MALAYSIA

## Abstract

Since 2013, epidemics caused by novel H7N9 avian influenza A viruses (AIVs) have become a considerable public health issue. This study investigated the evolution of these viruses at the population level. Compared to H7 and N9 before 2013, there were 18 and 24 substitutions in the majority of novel H7N9 AIVs, respectively. Nine of these in HA and six in NA were rare before 2013, and four of these in HA and two in NA displayed host tropism. S136(128)N and A143(135)V are located on the receptor binding sites of the HA1 subunit and might be important factors in determining the host species of novel H7N9 AIV. On an overall scale, the evolution of H7 and N9, both in terms of time distribution and host species, is under negative selection. However, both in HA and NA, several sites were under positive selection. In both the overall epidemics and the human-derived H7N9 AIVs, eight positive selection sites were identified in HA1, with some located within the known antigen epitopes or the receptor binding site(RBS) domain. This may induce variations in H7N9 AIV with positive selection. It is necessary to strengthen the surveillance of novel H7N9 AIVs, both in human and bird population to determine whether a new virus has emerged through selection pressure and to prevent future epidemics from occurring.

## Introduction

Influenza A viruses (IAVs) belong to the family Orthomyxoviridae. Based on the antigenic properties of the two surface glycoproteins hemagglutinin (HA) and neuraminidase (NA), IAVs are clustered into 18 HA (H1–H18) and 11 NA (N1–N11) subtypes [1]. Apart from H17, H18, N10, and N11, which were restrictively identified from bat samples in the form of H17N10 and H18N11, all of the other subtypes of the virus can circulate in avian species [2]. Occasionally, avian influenza A viruses (AIVs) can be transmitted from avian species to mammals, which may lead to the development of human pandemic strains by direct or indirect transmission [3,4].

In the spring of 2013, the first human infection with a novel AIV of H7N9 subtype (referred to as novel H7N9 AIV) emerged in Eastern China, causing severe human infections and deaths [5]. Subsequent outbreaks of H7N9 influenza in humans have occurred in the winter and spring of every year since then, and China has now witnessed six H7N9 epidemics [6–8]. According to statistics from the World Health Organization, there have been a total of 1,567 laboratory-confirmed cases of human H7N9 AIV infection, including 615 deaths, as of December 6, 2018 (http://www.fao.org/ag/againfo/programmes/en/empres/H7N9/situation_update.html). H7N9 has thus become a major public health issue. Previous studies have described the novel H7N9 AIV as a triple-reassortant virus of H7, N9, and H9N2 influenza viruses. Phylogenetic characteristics, variations involving key amino acids, virulence, and pathogenicity to human or animals regarding one or more strains isolated from human or bird have been well described [5–11], and numerous sequences have been submitted to public databases. These efforts have greatly facilitated research studies on the evolutionary dynamics of H7N9 AIVs based on large-scale sequence analysis.

Several viral proteins of IAVs are known to be responsible for immune response, host adaptation, or interspecies transmission, of which the membrane protein HA is the major determinant [12,13], while the balance between HA receptor-binding affinity and NA receptor-destroying activity is critical for the efficient growth of IAVs. NA also contributes to influenza virus species specificity and determines the virulence and drug resistance of AIVs [14]. In this study, we further clarified the molecular basis of host tropism and virulence characteristics of novel H7N9 AIVs and provide evidence on the surveillance and early warning signs of an H7N9 epidemic. We used relevant influenza virus bioinformatics databases to investigate the evolutionary dynamics of HA and NA, screen the key amino acid sites related to virulence and infectious characteristics, and predict the possible evolution and mutation trends of novel H7N9 AIVs in the future.

## Results

### Sequence distribution

As of August 29, 2018, there were 1,952 HA sequences of novel H7N9 AIVs in the two databases, of which 1,125 were derived from human and 827 from avian species. There were 1,918 NA sequences of novel H7N9 AIVs, of which 1,096 were derived from human and 822 from avian species. The main epidemic area of novel H7N9 is China.

For H7 and N9 before the H7N9 epidemics, 2,159 HA sequences were obtained from the two databases, of which 84 were derived from human and other mammals. A total of 758 NA sequences were obtained and except for one whale-derived isolate, all sequences were derived from birds. Because only a small number of sequences were derived from human and other mammals, H7 and N9 before the H7N9 epidemics were not further differentiated based on host.

### Substitutions of amino acid residues’ majorities of HAs and NAs

Compared to H7 before 2013, there were 18 characteristic substitutions in the majority of novel H7N9 AIV HA. Note that instead of deleting the signal peptide, parentheses were used to label the numbers according to the mature HA1 subunit of H3 (referred to A/Aichi/2/68(H3N2), Accession: EF614251). The substitutions were V11I, T130(122)A, D183(174)S, I188(179)V, G195(186)V, T198(189)A, I211(202)V, Q235(226)L, L245(236)M, D279(270)G, N307(298)D, E321(312)R, T410N, M427I, N455D, R462K, L506M, and A541V. Eleven of these are located in the HA1 subunit; some of these were even located in the receptor binding sites (RBS) or the known antigen epitopes A to E (Table 1).

**Table 1:** Substitutions and polymorphisms of amino acid composition of H7*.

Section			Site	[~, 2012] AV	[2013, ~] AV	[2013, ~] HU
**Signal peptide**			11	1108**V** 878C 28I 26M 9Y 7L 7T 5A 1E 1G 4? 85-	808**I** 9T 4V 6-	1092**I** 22T 2V 9-
**HA1**	**Antigen section**	**RBS**				
	A	120-loop	130(122)	1072**T** 922S 117A 8I 5P 3K 3R 1? 28-	731**A** 61T 19P 13S 1E 1V 1-	654**A** 413T 54P 2S 1E 1?
	B	120-loop	**136(128)**	2083**S** 44N 2G 1I 2? 27-	574**S** 247N 5D 1-	637**N** 484S 3D 1?
	A	right arm	**143(135)**	1825**A** 222T 69V 4P 5E 2K 1G 4? 27-	595**A** 221V 4L 4T 2P 1-	628**V** 483A 5T 3S 6?
	D		183(174)	1128**D** 923K 38G 36E 9A 2R 2N 1Y 20-	816**S** 8D 2N 1?	1109**S** 12N 1G 1R 2?
	D	180-helix	188(179)	1852**I** 265V 13T 5L 2M 1A 1N 20-	822**V** 5I	1117**V** 7I 1?
	B	180-helix	195(186)	1776**G** 274E 82V 3A 3D 1? 20-	806**V** 13A 8G	1089**V** 26A 4G 4I 2?
	B		198(189)	1573**T** 326S 115A 83D 38N 2I 1G 1E 1K 19-	822**A** 4T 1E	1122**A** 2V 1?
	D		211(202)	2115**I** 33V 11-	820**V** 7I	1125**V**
	D	left arm	235(226)	1863**Q** 1P 1E 1? 293-	747**L** 69Q 10I 1?	1038**L** 55Q 16I 5S 3H 2P 6?
			245(236)	905**L** 786M 441I 14V 4F 1? 8-	727**M** 91I 5I 4V	594**M** 474I 50L 6V 1?
			279(270)	969**D** 774G 294E 97S 18N 5R 1K 1?	822**G** 3A 2E	1112**G** 9R 3E 1?
	C		307(298)	2141**N** 15D 3S	822**D** 5N	1122**D** 2N 1G
	C		321(312)	1147**E** 606T 304K 64P 12R 9G 7S 5A 2D 1N 1I 1-	668**R** 151K 4G 4E	1031**R** 92K 1G 1?
**HA2**			**396**	1123**E** 660D 227G 14S 1? 134-	558**E** 267A 1P 1G	649**A** 472E 1G 1Q 2?
			410	1087**T** 491N 415S 17I 12G 137-	819**N** 6T 1H 1?	1106**N** 13S 3T 1D 1I 1?
			427	1884**M** 122I 10V 6L 137-	817**I** 7M 1L 1V 1?	1121**I** 3L 1?
			455	1722**N** 248S 31D 16T 3K 139-	810**D** 12N 4V 1-	1113**D** 6G 3N 3?
			462	1114**R** 906K 1? 139-	818**K** 8R 1-	1124**K** 1R
			**499**	1108**S** 891T 9R 5N 2A 2I 2? 140-	523**S** 269R 34N 1-	650**R** 470S 4N 1K
			506	895**L** 856M 263I 1V 2? 142-	820**M** 5I 1T 1-	1116**M** 7I 1L 1?
			541	1953**A** 35V 1T 1? 169-	815**V** 10A 1-	1119**V** 6A

*Note: Numbers in parentheses are according to the mature HA1 subunit of H3, referred to A/Aichi/2/68(H3N2), Accession: EF614251.

The composition ratios of amino acid polymorphisms revealed that nine substitutions, V11I, T130(122)A, D183(174)S, G195(186)V, T198(189)A, Q235(226)L, N307(298)D, E321(312)R, and N455D, were rare before 2013.

Compared to both HAs from before 2013 and from the avian-derived novel H7N9 AIVs, human-derived novel H7N9 significantly differed from them at site 136 (corresponding to site 118 of mature HA1 of H7 or site 128 of mature HA1 of H3), 143 (corresponding to site 125 of mature HA1 of H7 or site 135 of mature HA1 of H3), 396, and 499 (*p* < 0.05). The majority of human-derived AIVs at these sites are N, V, A, and R, respectively, but those derived from avian species are S, A, E, and S, respectively. However, the substitution Q235(226)L did not show any host specificity, and the compositions of L occupied 92.27% of human-derived and 90.33% of avian-derived isolates (Table 1).

Compared to N9 before 2013, a total of 24 substitutions were identified in novel H7N9 AIVs. The composition ratios of amino acid polymorphisms displayed six substitutions, T19A, N40G, E84(79, referred to A/Shanghai/02/2013(H7N9), Accession: NC_026429) that characterized by a deletion located from 69–74 amino acid sites)N, D112(107)S, G359(354)A, and T401(396)A, which were rare before 2013. Similarly, S247(242)P and N327(322)S showed significant differences (*p* < 0.05) between human- and avian-derived N9 compared to both isolates established before 2013 and novel H7N9 AIVs. However, no substitution occurred at known drug resistance sites or at known potential glycosylation sites (Table 2).

**Table 2:** Substitutions and polymorphisms of amino acid composition of N9*.

Site	[~, 2012] AV	[2013, ~] AV	[2013, ~] HU
16	678**V** 34I 8A 1? 51-	531**I** 242T 3V 1R 1? 44-	678**T** 413I 4V 1A
19	516**T** 153I 54A 1M 48-	780**A** 6T 36-	1085**A** 7T 2V 1R 1?
40	400**N** 305S 18G 13D 1I 1T 34-	767**G** 20S 1? 34-	1065**G** 24S 6D 1?
50	533**A** 147T 49E 13G 4S 2V 2I 22-	752**T** 32I 5A 33-	1054**T** 30I 11A 1K
53	510**A** 236T 2V 1S 1G 22-	786**T** 2I 1A 33-	1085**T** 9P 1M 1I
80(75)	398**K** 346R 4G 2S 1E 21-	782**R** 6K 3S 31-	1090**R** 4K 1G 1S
81(76)	723**A** 14T 6E 2S 2P 2I 1V 1? 21-	782**T** 5A 4K 31-	1088**T** 5K 1R 2?
84(79)	493**E** 199G 45D 10S 2V 2N 21-	772**N** 10D 8G 1S 31-	1069**N** 15S 6D 2K 1Y 1I 2?
112(107)	379**D** 317N 54S 1K 21-	811**S** 11N	1089**S** 7N
189(184)	467**R** 297K 8-	821**K** 1E	1096**K**
190(185)	517**A** 231S 13T 3F 8-	821**S** 1F	1095**S** 1Y
207(202)	397**I** 360V 15-	788**V** 34I	1047**V** 49I
215(210)	533**T** 223A 1K 15-	805**A** 14T 2V 1S	1089**A** 4V 3T
**247(242)**	753**S** 2P 1Y 16-	559**S** 263P	619**P** 473S 3H 1?
253(248)	539**E** 215D 1K 1H 16-	815**D** 6E 1C	1085**D** 5E 4N 2Y
256(251)	462**V** 294I 16-	819**I** 3V	1096**I**
269(264)	389**P** 363S 3Y 1A 1? 15-	818**S** 2Y 1P 1?	1093**S** 3P
286(281)	480**Q** 271R 5K 2E 1D 13-	822**R**	1087**R** 6Q 3L
287(282)	483**A** 256T 15V 3S 2K 13-	822**T**	1090**T** 4A 2K
**327(322)**	760**N** 12-	403**N** 252S 167T	624**S** 393N 79T
334(329)	295**A** 225N 220T 11V 3S 2I 1M 1H 1D 1? 12-	817**N** 2T 2D 1S	1071**N** 11D 8S 4K 2T
335(330)	592**V** 168I 12-	807**I** 10V 5M	1030**I** 54M 9V 2L 1T
359(354)	430**G** 223V 48D 25A 16S 10L 5I 2F 1E 12-	782**A** 29D 5T 5V 1F	982**A** 56T 49D 7V 1G 1?
392(387)	508**T** 245I 4V 2A 1M 12-	809**I** 3S 3T 2V 5-	1084**I** 10V 2T
401(396)	760**T** 12-	798**A** 16T 3D 5-	1049**A** 26T 9V 7S 5D
418(413)	507**E** 251D 1V 1? 12-	816**D** 6-	1095**D** 1N

*Note: Numbers in parentheses are according to Refseq A/Shanghai/02/2013(H7N9), Accession: NC_026429, which characterized by a deletion located from 69–74 amino acid sites).

### Spatial structure analyses of HA1 subunits

Homology modeling for the tertiary structure demonstrated that sites 136 and 143 are both located on the surface of the HA1 subunit. Site 136 is located within the head of the HA1 subunit and adjacent to the 120-loop of RBS domain, whereas site 143 is situated within the left arm (or left side) of the RBS.

**Fig 1:**
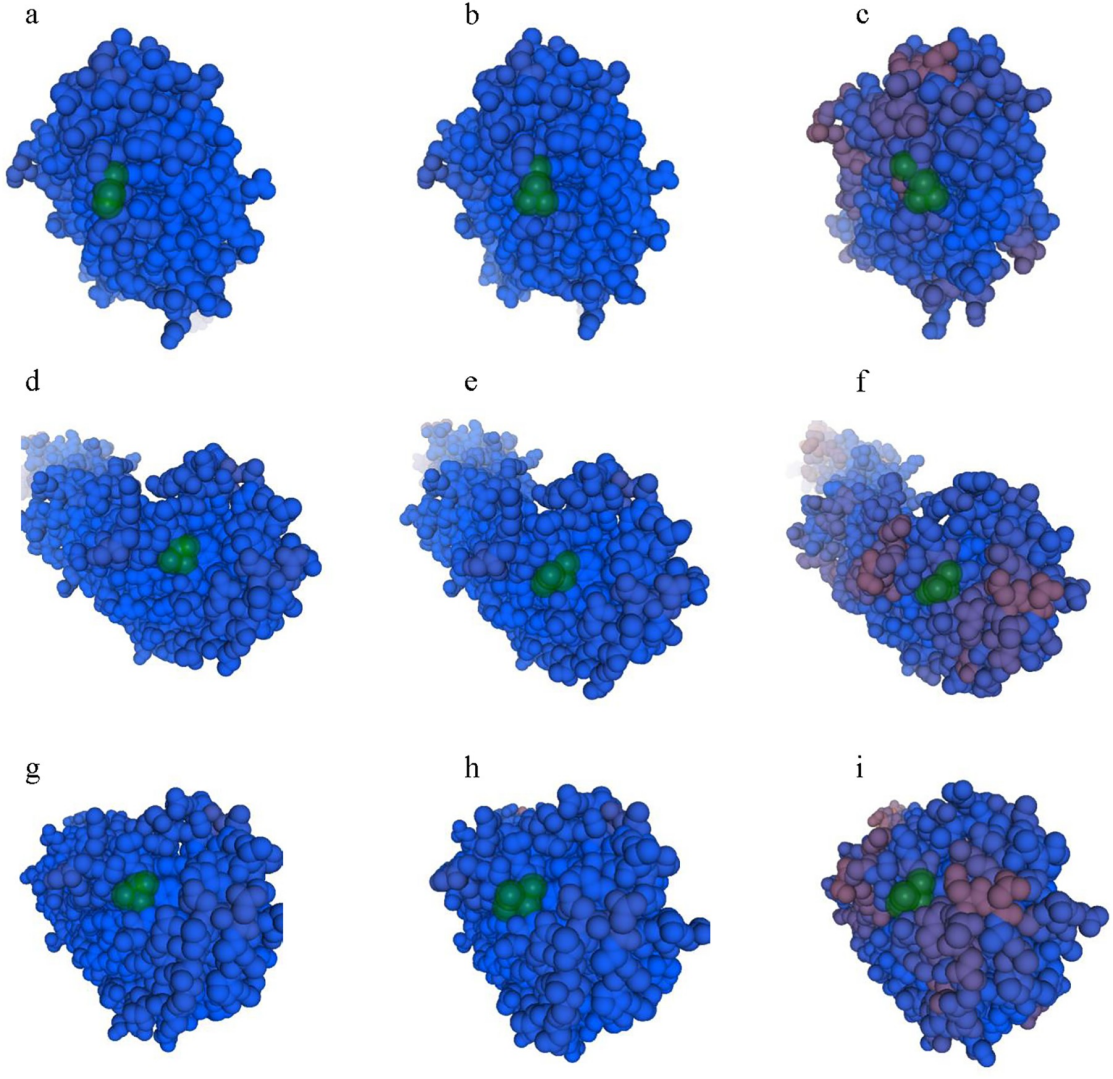
Spatial structures of HA1 belonging to the majorities of avian- and human-derived H7N9 AIVs and the transitional ones. The focused substitutions were labelled by deep green. a, d, and g are of the majority of avian-derived H7N9 AIVs, and the template for homology modelling was 4fqv (Identity 98.44%); b, e, and h are of the transitional ones, and the template for homology modelling was also 4fqv (Identity 98.13%); c, f, and i are of the majority of human-derived H7N9 AIVs, and the template for homology modelling was 5v2a (Identity 99.07%). a-c display the substitutions of site 128(136), while d-i displayed the substitutions of site 135(143) from two different perspectives. Other information refers to S1 File.

A substitution from serine (S) of avian-derived H7 [which is represented by A/chicken/Wuxi/0405005G/2013(H7N9) with 100% identity to the avian-derived majority] to asparagine (N) of human-derived one [which is represented by A/Quzhou/1/2015(H7N9) with 99% identity to human-derived majority] at site 136 results in a more prominent bump in the 120-loop of RBS domain. Moreover, the larger side chain of N also provides a wider contact area for the binding of RBS to receptors located in host epithelial cells. This change in spatial structure can be achieved by modifying only a single site, i.e., S136N, of the transitional HA1 (Fig 1a–1c and S1 File).

For site 143, a substitution from alanine (A) to valine (V) leads to a greater change in the tertiary structure of the RBS domain. However, this conformational change is apparently not caused by a single amino acid residue, A143V, but seems to involve a series of amino acid residue substitutions. These result in a smaller and shallower pocket in the RBS domain (Fig 1d–1i). This means that it is more difficult for the viruses to bind to receptors on the surface of the host epithelial cells. On the contrary, it will be easier for the viruses to dissociate from the receptors. For viruses that have infected host cells and successfully replicated in large quantities, this conformation will be beneficial for the release of viruses from surfaces of host cells, and thus for the rapid proliferation of viruses. This may partly explain why novel H7N9 AIV has higher virulence to human and bird hosts than previous H7 subtypes AIVs.

### Positive selection sites in H7 and N9

Matrices, including 105, 66, 105, and 112 H7 sequences, and 81, 61, 107, and 120 N9 sequences were selected and used in the analysis of selection pressure according to time distribution and host species (S2 File).

Overall scale selection pressure analysis showed that ɷ values of all H7 before 2013, all novel H7N9 AIVs, human-derived H7N9 AIVs and avian-derived H7N9 AIVs were 0.26, 0.19, 0.23, and 0.16, respectively, while these values of N9 were 0.29, 0.19, 0.21, and 0.13, respectively. All ɷ values on the overall scale were < 1.

Analysis of single amino acid sites one-by-one revealed that two sites of HA1 subunits belonging to the H7 subtype AIVs before 2013 were affected by positive selection pressure, namely, 284(275) and 334(325). None of these sites formed a *de facto* substitution. Similarly, among the 12 positive selection sites of NA belonging to the N9 subtype AIVs before 2013, only variations at site 50 and 287(282) may be caused by positive selection pressure. It is particularly worth noting that since the emergence of the H7N9 epidemic in China in 2013, many positive selection sites within H7 have appeared in both the overall epidemics and the human-derived H7N9 AIVs. Most of these even located within the HA1 subunit. For the overall epidemics and human-derived AIVs, there were both eight positive selection sites, some of them were located within known antigen epitopes or the RBS domain (Table 3).

**Table 3:** Positive selection pressure sites within H7 and N9 sequences.

Protein			[~, 2012] AV	[2013, ~] H7N9	[2013, ~] HU	[2013, ~] AV
**Signal peptide**			3,4	3,4,6,7,12,14	3,7,12,14	4,6
**HA1**	**Antigen section**	**RBS**				
	C				56(48)	
	E				65(57)	
	E				67(59)	
	A	120-loop		130(122)		
	A	right arm		143(135)	143(135)	143(135)
	A			148(140)	148(140)	148(140)
	D	Left arm		231(222)	231(222)	
	D	Left arm		235(226)	235(226)	235(226)
				276(267)	276(267)	
	C		284(275)			
	C			285(276)		
	C			321(312)		321(312)
			334(325)			
**HA2**			553,555,556,557	540,556,557		469,557,558
**NA**			2,4,6,45,50,51,287,461,462,464,465,467	6,16,22,46,50,82,84,252,270,400	16,18,22,46,270,293,309	6,16,22,84,238,241

Other positive selection sites within both H7 and N9 and in terms of time distribution and host species are listed in Table 3.

## Discussion

In this study, based on large-scale bioinformatics analyses for H7 and N9 nucleotide sequences belonging to novel H7N9 AIVs, H7, and N9 subtypes IAVs before 2013, the origin and evolution of novel H7N9 were discussed, and the future evolution of novel H7N9 was also reasonably predicted.

Human infections and deaths caused by novel H7N9 AIVs are much higher than any previous H7 subtype influenza [15,16]. Compared with early H7, the HA of the novel H7N9 AIVs appeared to have as many as 18 substitutions. Many of these substitutions are located in known antigenic epitopes or the RBS domain of HA1 and were rare in previous H7 subtype AIVs. This means that such substitutions are likely to play a pivotal role in the emergence of novel H7N9 AIV and the outbreak of epidemics; one or more of these substitutions work together might be the molecular basis of virulence enhancement. As for NA, its role is mainly catalytic and it is also important for drug resistance. However, compared with N9, most of the substitutions within NA of novel H7N9 AIVs are rarely located on known drug resistance sites or enzyme activity centers. There are also many sites that are not located at known key sites such as 245(236) and 279(270) in the HA1 subunit and all substitutions involving HA2. There are two explanations for such substitutions. One is that although some amino acid sites are related to virulence and pathogenicity of the virus, these have not been confirmed to date. For example, because HA2 is not a membrane surface subunit of HA, its role in virulence and pathogenicity has been neglected for a long time. In fact, as non-surface proteins, PB1, PB2, and M are all related to the pathogenicity and host tropism of AIVs [16–18], and new antigenic epitopes of novel H7N9 AIVs also have been frequently reported [19]. Second, some viral characteristics do not function through a single substitution, but probably through the synergy of multiple substitutions [16,20]. Therefore, the significance of these substitutions in virulence and pathogenicity deserves further investigation.

As typical AIVs, spillover infections of H7 or N9 from birds to humans should have a corresponding molecular basis. This study found that there are four significant substitutions between avian-derived and human-derived H7 HAs, namely, 136(128), 143(135), 396, and 499. Two significant substitutions in N9 NAs, namely 247(242) and 327(322), also display host specificity. Whether these substitutions are more conducive to cross-species transmission of the virus require further investigation, although homology modelling has revealed that 136(128) and 143(135) are indeed located within the RBS domain of HA1 [21,22] and could alter the spatial structure of this domain alone or in cooperation with other sites. These substitutions might be important to determine which host species can be infected by these H7N9 AIVs and play a decisive role in H7N9 spillover infections from bird to human. So far, there is no report on the substitution of these two sites and thus these have yet to be confirmed. However, a Q235(226)L substitution, which corresponds to position 226 in H3N2 subtype HA and its substitution is considered to be the key factor causing cross-species transmission of AIVs[23–25], emerged in both majorities of human- and avian-derived novel H7N9 AIVs, and the differences were significant (*p* < 0.05) compared to H7 before 2013, but within novel H7N9 epidemics, these did not show any host specificity as reported in some studies [5,18]. This suggests that Q235(226)L may be utilized in determining the virulence and pathogenicity of novel H7N9 AIVs, but it does not necessarily determine the host type of H7N9.

Analysis of selection pressure may suggest whether a mutation is supported or hindered by natural selection, the direction of virus evolution, and possible amino acid sites involved [26,27]. On an overall scale, the evolution of H7 and N9, both in terms of time distribution and host species, is under negative selection pressure, which is consistent with previous research results on other subtypes IAVs [27–30]. Examination of single amino acid sites in AIVs before 2013 or in novel H7N9 AIVs or in avian-derived or human-derived H7N9 AIVs has revealed multiple sites on H7 and N9 that are under positive selection pressure. Only very few positive selection sites formed a *de facto* substitution. This indicates that in the evolutionary process of the novel H7N9 AIVs, purifying selection still played the leading role. Since the emergence of the H7N9 epidemic in China in 2013, more positive selection sites have appeared in overall epidemics and/or human-derived H7N9 AIVs, e.g., 143(135), 148(140), 231(222), 235(226), 276(267)/56(48), 65(57), 67(59), 130(122), 285(276), and 321(312), which are located within the HA1 subunit. These findings are consistent with several recent findings. For example, Xiang et al. and Fang et al. also reported that sites within HA or NA are undergoing positive selection [31,32]. This suggests that H7N9 may rapidly vary under the positive selection pressure. It is necessary to strengthen the surveillance of novel H7N9 AIVs, especially those isolated from human, to determine whether a new virus has emerged through selection pressure and to prevent future epidemics from occurring.

Selection pressure may be manifested by the way of host immune system response to the virus. Although the H7N9 vaccine has not been used in humans, with the increase in natural infections in the population, the immune background of human population gradually accumulates and the immune barrier is gradually improving [26,33]. This inference is in line with reality; in China, especially in East China, there are relatively high proportions of antibodies against H7 subtype AIVs in human, particularly, in the poultry workers [34,35]. In addition, as H7N9 vaccines have been widely used to prevent and control avian influenza in poultry farms [8,36], the immune pressure among poultry will increase over time, and the viruses will further evolve. Thus, it is also of great significance to strengthen the continuous surveillance of H7N9 AIV poultry to better understand the mutational pattern of the virus, develop prevention and control measures, and screening vaccine strains.

## Conclusions

Compared with early H7 and N9, the HA and NA of the novel H7N9 AIVs appeared to have as many as 22 and 26 substitutions, respectively. Four sites within H7, i.e., S136(128)N, A143(135)V, E396A, and S499R, and two sites within N9, i.e., S247(242)P and N327(322)S, showed significant host specificity, which may play a role in determining host tropism. To elucidate the mechanism of the novel H7N9 epidemic occurrence and transmission, further experiments are needed to confirm these findings. This study also found that in the early evolution of H7 and N9, positive selection pressure played a limited role, and purifying selection largely contributed to its pathogenicity. However, since the outbreak of H7N9, the virus has been subjected to positive selection pressure at many sites, especially in human population. This suggests that H7N9 may rapidly vary under the positive selection pressure. It is necessary to strengthen the surveillance of novel H7N9 AIVs both in human and bird populations to determine whether a new virus has emerged through selection and to prevent future epidemics.

## Materials and methods

### Sequences preparation

HA and NA nucleotide sequences were downloaded from two databases, the Influenza Virus Resource of NCBI (http://www.ncbi.nlm.nih.gov/genomes/FLU/aboutdatabase.html) and the Global Initiative on Sharing Avian Influenza Data (GISAID, http://platform.gisaid.org/epi3/frontend), on August 29, 2018. When downloading from GISAID, the option of *only GISAID uploaded isolates* was chosen. For novel H7N9 AIVs, matrices of both HA and NA were downloaded as one of the following: from all host animals, from human only, and from avian only. The *collection date* (not the *release date* or *submission date*) was set as *2013/03/01-*. For the H7 and N9 before the H7N9 epidemics, sequence matrices were downloaded as the formula of H7Nx and HxN9, and the *collection date* (not the *release date* or *submission date*) was set as –*2012/12/31*. After removing repetitive sequences manually according to the isolates’ name and removing poor quality sequences that were filled with consecutive letters “n”, all of the matrices were aligned by MAFFT v7.0 (http://mafft.cbrc.jp) and trimmed by MEGA 5.1 (https://www.megasoftware.net/). Only the codon of each sequence remained in the matrices.

### Analyses on variation and polymorphism of amino acid site

Using the MegAlign module in the Lasergene v7.1 software (https://www.dnastar.com), nucleotide sequences in each matrix were translated into amino acid sequences, and the amino acid majority at each site was then calculated. The amino acid majorities of HA and NA belonging to the novel H7N9 AIVs and H7Nx or HxN9 that were established before 2013 were again compared using MegaAlign. When there were different majorities at the same sites, their polymorphisms of amino acids were counted by MS-word by using the function of search-replacement. The composition ratios of amino acid polymorphisms were tested by chi-square analysis with SPSS 13.0. The difference was significant when *p* < 0.05. The known functional sites such as antigen epitopes or receptor binding site (RBS) were based on H3N2 IAVs and other reports regarding H7, N9, and H7N9 IAVs [37–41].

### Spatial structure analyses on HA1 subunits

As mentioned above, HA is the major determinant for host immune response, host adaptation, or interspecies transmission. The globular head of HA, namely HA1, has the most important domain for binding to receptors on host epithelial cells triggering viral infection, as well as for antigen-antibody reactions [12,13]. The variations in the amino acids of the protein primary structure at key sites might lead to changes in protein tertiary structure, which allows ease or difficulty for viruses to bind to specific ligands such as antibodies or receptors located on the surfaces of host-specific epithelial cells. Amino acid sequences of HA1 most similar to human- or avian-derived majorities were obtained by BLASTp (https://blast.ncbi.nlm.nih.gov/Blast.cgi), while the transitional form of HA1 was obtained by manually modifying the specific sites on the avian-derived majority ones. Homology modelling was done by Automated Mode via the ExPASy web server (https://swissmodel.expasy.org)[42,43].

### Selection pressure analysis of protein-encoded genes

For a protein-encoded nucleotide sequence, if the nonsynonymous substitution caused by a codon mutation is significantly greater than the synonymous substitution caused by this mutation, then this amino acid site is considered subject to a positive selection pressure; if the situation is reversed, then it is considered to be subjected to negative selection pressure. Positive selection pressure may be beneficial to the survival of new mutations, while negative selection pressure may lead to the elimination of new mutations. Selection pressure can be expressed by formula ɷ = *dN/dS*, which is the ratio of nonsynonymous changes per nonsynonymous site (*dN*) to synonymous changes per synonymous site (*dS*). This ratio is used to infer the type of evolutionary pressure acting on a protein: when ɷ>1, positive Darwinian selection is inferred, when ɷ = 1.0, strict neutrality is inferred, and when ɷ<1, purifying selection is inferred [44].

The overall selection pressures within each matrix of HA or NA were calculated by using the Datamonkey online server (http://www.datamonkey.org/). HKY85 was selected as the phylogeny test model and single likelihood ancestor counting (SLAC) served as the algorithm. When prior trees were needed, neighbor-joining statistical method, Kimura-2 parameters model, and the bootstrap test with 1,000 replicates were adopted to construct the trees[28,45]. Positive selection pressure analyses for each amino acid site one-by-one were calculated by MEGA5.10. Prior trees were also constructed by the methods mentioned above. *p*≦0.20 was determined as a statistically significant difference [46–48]. Due to the greater accumulation of deleterious mutations that have not yet purged at the population level, estimates of selection pressure can be biased when analyzing intensively sampled populations. To avoid bias, repeatedly submitted sequences were strictly eliminated. Based on the phylogenetic tree and homogeneous identities, sequences shared more than 99% identity were randomly left one for subsequent analysis, and the rest were removed.

## Supporting information

S1 File Amino acid sequences used for homology modelling in this study. (DOCX)

S2 File Nucleotide sequences used for the analysis of selection pressure in this study. (DOCX)
