# Supplementary material for: Evolutionary dynamics of the H7N9 avian influenza virus based on large-scale sequence analysis
Source: PLoS One. 2019 Aug 12;14(8):e0220249. doi: 10.1371/journal.pone.0220249 (PMC6690514; doi:10.1371/journal.pone.0220249)
Supplement: S1 File — (DOCX) [file pone.0220249.s001.docx]

**S1 File.** Amino acid sequences used for homology modelling in this study

Amino acid sequences 2, 3, and 5 were used for homology modelling. The information is listed as below:

1. The majority of HA1 belonging to avian-derived H7N9 AIVs

DKICLGHHAVSNGTKVNTLTERGVEVVNATETVERTNIPRICSKGKRTVDLGQCGLLGTITGPPQCDQFLEFSADLIIERREGSDVCYPGKFVNEEALRQILRESGGIDKEAMGFTYSGIRTNGATSACRRSGSSFYAEMKWLLSNTDNAAFPQMTKSYKNTRKSPALIVWGIHHSVSTAEQTKLYGSGNKLVTVGSSNYQQSFVPSPGARPQVNGLSGRIDFHWLMLNPNDTVTFSFNGAFIAPDRASFLRGKSMGIQSGVQVDANCEGDCYHSGGTIISNLPFQNIDSRAVGKCPRYVKQRSLLLATGMKNVPEIPKGR

2. HA1 of A/chicken/Wuxi/0405005G/2013(H7N9) (Sequence ID: AKM15203)

DKICLGHHAVSNGTKVNTLTERGVEVVNATETVERTNIPRICSKGKRTVDLGQCGLLGTITGPPQCDQFLEFSADLIIERREGSDVCYPGKFVNEEALRQILRESGGIDKEAMGFTYSGIRTNGATSACRRSGSSFYAEMKWLLSNTDNAAFPQMTKSYKNTRKSPALIVWGIHHSVSTAEQTKLYGSGNKLVTVGSSNYQQSFVPSPGARPQVNGLSGRIDFHWLMLNPNDTVTFSFNGAFIAPDRASFLRGKSMGIQSGVQVDANCEGDCYHSGGTIISNLPFQNIDSRAVGKCPRYVKQRSLLLATGMKNVPEIPKGR

This amino acid sequence shared 100% identity with sequence 1, and was used as the representative for majority of HA1 belonging to avian-derived H7N9 AIVs.

3. The transitional HA1 between avian- and human-derived majorities of H7N9 AIVs

DKICLGHHAVSNGTKVNTLTERGVEVVNATETVERTNIPRICSKGKRTVDLGQCGLLGTITGPPQCDQFLEFSADLIIERREGSDVCYPGKFVNEEALRQILRESGGIDKEAMGFTYNGIRTNGVTSACRRSGSSFYAEMKWLLSNTDNAAFPQMTKSYKNTRKSPALIVWGIHHSVSTAEQTKLYGSGNKLVTVGSSNYQQSFVPSPGARPQVNGLSGRIDFHWLMLNPNDTVTFSFNGAFIAPDRASFLRGKSMGIQSGVQVDANCEGDCYHSGGTIISNLPFQNIDSRAVGKCPRYVKQRSLLLATGMKNVPEIPKGR

This sequence was obtained from manually modified sequences 1 and 2 at two sites (red labelled) by using the amino acid residues of sequence 4 and 5.

4. The majority of HA1 belonging to human-derived H7N9 AIVs

DKICLGHHAVSNGTKVNTLTERGVEVVNATETVERTNIPRICSKGKRTVDLGQCGLLGTITGPPQCDQFLEFSADLIIERREGSDVCYPGKFVNEEALRQILRESGGIDKEAMGFTYNGIRTNGVTSACRRSGSSFYAEMKWLLSNTDNAAFPQMTKSYKNTRKSPALIVWGIHHSVSTAEQTKLYGSGNKLVTVGSSNYQQSFVPSPGARPQVNGLSGRIDFHWLMLNPNDTVTFSFNGAFIAPDRASFLRGKSMGIQSGVQVDANCEGDCYHSGGTIISNLPFQNIDSRAVGKCPRYVKQRSLLLATGMKNVPEIPKGR

5. A/Quzhou/1/2015(H7N9) (Sequence ID: AKI82233)

DKICLGHHAVSNGTKVNTLTERGVEVVNATETVERTNIPRICSKGKRTVDLGQCGLLGTITGPPQCDQFLEFSADLIIERREGSDVCYPGKFVNEEALRQILRESGGIDKEAMGFTYNGIRTNGVTSACRRSGSSFYAEMKWLLSNTDNAAFPQMTKSYKNTRKSPAIIVWGIHHSVSTAEQTKLYGSGNKLVTVGSSNYQQSFVPSPGARPQVNGLSGRIDFHWLMLNPNDTVTFSFNGAFIAPDRASFLRGKSMGIQSGVQVDANCEGDCYHSGGTIISNLPFQNIDSRAVGKCPRYVKQRSLLLATGMKNVPEIPKGR

This sequence shared 99% identity with sequence 4, and was used as the representative for majority of HA1 belonging to human-derived H7N9 AIVs.
