# Supplementary material for: Evolutionary dynamics of the H7N9 avian influenza virus based on large-scale sequence analysis
Source: PLoS One. 2019 Aug 12;14(8):e0220249. doi: 10.1371/journal.pone.0220249 (PMC6690514; doi:10.1371/journal.pone.0220249)
Supplement: S2 File — (DOCX) [file pone.0220249.s002.docx]

**S2 File.** Nucleotide Sequences used for the analysis of selection pressure in this study

1. **H7 before 2013** (105 sequences)

EPI492521 EPI304483 EPI210106 EPI372394 EPI860880 EPI169519 EPI441604 EPI492519 EPI356351

KU289738 CY138769 CY075941 FN386467 CY035692 CY035696 CY125365 EU684260 CY185969 GQ921322 AY303633 V01105 AF202239 KF493206 HM346477 KY676326 CY022701 CY095036 CY024786 EF675618 DQ525411 AF202228 GU052907 KF258993 JQ988864 U20465 CY006029 KF258981 JN244240 AB828685 AB268557 AB450448 AB558255 AB297923 AB269695 GQ221699 JX307201 CY061610 KX061808 EU980454 Z12617 AF202237 CY130142 KF258958 L43915 CY094461 CY035587 CY034238 JX080762 AF202250 CY120555 CY177045 CY107853 JX444835 GU053270 KR862488 CY061618 CY021621 AY999987 GU053143 KF695354 CY178855 MF147461 KX978740 AF202240 AB517632 KY644296 CY187037 CY076293 GU052954 KT777909 AB538458 AB538459 AF072399 AY240924 JX523347 CY185895 CY005981 FJ610056 CY005980 CY185829 CY103015 GU052961 M17736 AB269872 CY107860 CY015065 KF493477 CY028676 AF202246 AB269693 AB269692 JN244233 FJ750874 KC609769 KC876683

1. **N9 before 2013** (81 sequences)

EPI1139885

AB289342 AB472038 AB569553 AB719900 AB807771 CY003857 CY004361 CY014788 CY014808 CY020967 CY021871 CY042594 CY054301 CY060192 CY077586 CY077654 CY081374 CY094183 CY094295 CY094881 CY097153 CY109684 CY116777 CY117205 CY122001 CY125351 CY125744 CY126682 CY127640 CY129263 CY136874 CY139371 CY165654 CY166623 CY166762 CY176952 CY177704 CY177728 CY178905 CY179365 CY184009 CY184626 CY186865 CY186905 CY203711 EU429798 EU523137 EU742646 GQ257427 GQ257489 GU051967 HM849012 HQ244409 HQ541731 JN244223 KC599300 KC790087 KF259697 KF259700 KF259712 KF259715 KF259718 KF259724 KF259725 KF445413 KF695330 KF771199 KF772950 KJ161965 KJ525971 KR824553 KR824740 KX977684 LC339653 MF146170 MF147343 MH071484 MH501395 MH597013 MH597818

1. **Avian-derived H7 of H7N9** (66 sequences)

EPI1010107 EPI1054126 EPI1054142 EPI1055400 EPI1055432 EPI580327 EPI659383 EPI759897 EPI918744 EPI918897 EPI918953 EPI919067 EPI919075 EPI919123 EPI919139 EPI919147 EPI979549

KC899669 KM879322 KP185945 KP413523 KP413907 KP414891 KP415444 KP415858 KP415900 KP417325 KP417502 KP418208 KP418224 KP657909 KP765989 KP766004 KP766008 KP766035 KP766048 KP766049 KP766054 KP766062 KP766065 KP766072 KP766073 KP766077 KP864451 KU143280 KY751040 KY751066 MF629989 MF630245 MF630261 MF630293 MF630309 MF630325 MF630333 MF630373 MF630389 MF630405 MF630445 MF630453 MF630469 MF630501 MF630509 MF630533 MF950910 MF950911 MG739458

1. **Avian-derived N9 of H7N9** (61 sequences)

EPI1010109 EPI1054125 EPI528369 EPI580349 EPI665636 EPI666284 EPI917104 EPI918722 EPI918781 EPI918923 EPI918947 EPI919085 EPI919527 EPI979516 EPI979548

KF259730 KP185531 KP413267 KP414050 KP415583 KP415885 KP455980 KP657911 KP766136 KP766149 KP766150 KP766155 KP766156 KP766161 KP864453 KP864454 KU143379 KY221846 KY751112 KY751113 KY751128 KY751131 KY751132 KY855520 KY855528 MF184017 MF280192 MF629951 MF629967 MF630031 MF630111 MF630199 MF630207 MF630247 MF630311 MF630327 MF630335 MF630343 MF630447 MF630455 MF630471 MF630487 MF630495 MF630511 MF630519 MF630535

1. **Human-derived H7 of H7N9** (105 sequences)

EPI1013273 EPI1022665 EPI1075371 EPI1100423 EPI1100447 EPI1100479 EPI1100519 EPI1100655 EPI1100767 EPI1101455 EPI1101567 EPI1101575 EPI1101583 EPI1101607 EPI1101615 EPI1101623 EPI1101663 EPI1101679 EPI1101847 EPI1101919 EPI1101935 EPI1102007 EPI1102174 EPI1102261 EPI1102309 EPI1102333 EPI1102469 EPI1102581 EPI1102605 EPI1102789 EPI1102869 EPI1102901 EPI1102917 EPI1102965 EPI1102973 EPI1103364 EPI1103831 EPI1103887 EPI1103895 EPI1103911 EPI1103951 EPI1103967 EPI1104023 EPI1252151 EPI439486 EPI445912 EPI451254 EPI509120 EPI510157 EPI531793 EPI566036 EPI566044 EPI566116 EPI566124 EPI576028 EPI627129 EPI627145 EPI627161 EPI627249 EPI627377 EPI627481 EPI627553 EPI627633 EPI627681 EPI627808 EPI627928 EPI628056 EPI628080 EPI628208 EPI628360 EPI628568 EPI628808 EPI630218 EPI655902 EPI656156 EPI656195 EPI656306 EPI656370 EPI656418 EPI656442 EPI656506 EPI756011 EPI759847 EPI866545 EPI866569 EPI872958 EPI884222 EPI887708 EPI887812 EPI887836 EPI887868 EPI922374 EPI926825 EPI926857 EPI930815 EPI971108 EPI971276 EPI971300 EPI997217

KF150616 KF500925 KP414178 KP416614 KP864443 MF370259

1. **Human-derived N9 of H7N9** (107 sequences)

EPI1013256 EPI1057927 EPI1075370 EPI1075386 EPI1100462 EPI1100510 EPI1100518 EPI1100766 EPI1100782 EPI1101446 EPI1101454 EPI1101478 EPI1101526 EPI1101558 EPI1101566 EPI1101574 EPI1101582 EPI1101606 EPI1101646 EPI1101654 EPI1101670 EPI1101686 EPI1101926 EPI1102006 EPI1102078 EPI1102195 EPI1102324 EPI1102468 EPI1102548 EPI1102748 EPI1102844 EPI1102868 EPI1102964 EPI1102972 EPI1102996 EPI1103004 EPI1103846 EPI1103894 EPI1103910 EPI1103982 EPI1104006 EPI1252150 EPI439487 EPI447730 EPI451255 EPI451274 EPI451333 EPI460769 EPI477451 EPI531784 EPI533245 EPI534116 EPI534297 EPI556841 EPI557131 EPI559416 EPI566043 EPI566051 EPI566075 EPI626984 EPI627128 EPI627184 EPI627240 EPI627368 EPI627376 EPI627640 EPI627672 EPI627807 EPI627839 EPI627863 EPI628055 EPI628079 EPI628207 EPI628367 EPI628503 EPI628567 EPI628615 EPI628783 EPI628815 EPI631996 EPI632010 EPI656393 EPI656401 EPI656417 EPI656497 EPI759867 EPI866568 EPI887739 EPI887811 EPI887843 EPI887979 EPI888067 EPI926824 EPI926840 EPI971107 EPI971227 EPI971307 EPI971523

KC853231 KJ195794 KJ195799 KJ946418 KP864441 KP864447 KP864449 MF370256 MF370264

1. **Hu & Av H7 of H7N9** (112 sequences)

EPI1013273 EPI1022665 EPI1054126 EPI1054142 EPI1055400 EPI1055432 EPI1075371 EPI1100423 EPI1100655 EPI1101447 EPI1101455 EPI1101495 EPI1101567 EPI1101583 EPI1101607 EPI1101615 EPI1101623 EPI1101663 EPI1101935 EPI1102174 EPI1102261 EPI1102309 EPI1102469 EPI1102581 EPI1102869 EPI1102901 EPI1102917 EPI1102965 EPI1102973 EPI1103831 EPI1103887 EPI1103895 EPI1103967 EPI1104023 EPI1252151 EPI439486 EPI451254 EPI510157 EPI531793 EPI566044 EPI566116 EPI566124 EPI580327 EPI627129 EPI627145 EPI627249 EPI627377 EPI627481 EPI627497 EPI628056 EPI628080 EPI630218 EPI656418 EPI656442 EPI656506 EPI659383 EPI759897 EPI866569 EPI866577 EPI872958 EPI884222 EPI887708 EPI887812 EPI887836 EPI918897 EPI919123 EPI919139 EPI922374 EPI926825 EPI926857 EPI930815 EPI971276

KC899669 KP185945 KP414891 KP415444 KP415858 KP415900 KP417502 KP418208 KP418224 KP657909 KP766008 KP766035 KP766048 KP766049 KP766054 KP766062 KP766065 KP766072 KP766073 KP766077 KP864451 KU143280 KY751040 MF629989 MF630245 MF630261 MF630293 MF630309 MF630325 MF630333 MF630373 MF630389 MF630405 MF630445 MF630453 MF630469 MF630501 MF630509 MF630533 MG739458

1. **Hu & Av N9 of H7N9** (120 sequences)

EPI1057927 EPI1075370 EPI1075386 EPI1100462 EPI1100510 EPI1100782 EPI1101446 EPI1101454 EPI1101478 EPI1101526 EPI1101574 EPI1101582 EPI1101606 EPI1101646 EPI1101654 EPI1101670 EPI1101686 EPI1101926 EPI1102078 EPI1102195 EPI1102468 EPI1102748 EPI1102868 EPI1102964 EPI1102972 EPI1102996 EPI1103004 EPI1103846 EPI1103894 EPI1103910 EPI1103982 EPI1104006 EPI1252150 EPI447730 EPI451255 EPI451274 EPI451333 EPI528369 EPI531784 EPI557131 EPI566043 EPI580349 EPI626984 EPI627128 EPI627240 EPI627368 EPI627376 EPI627640 EPI627807 EPI627863 EPI628055 EPI628079 EPI628367 EPI628615 EPI628751 EPI631996 EPI656393 EPI656417 EPI656497 EPI665636 EPI666284 EPI759846 EPI759867 EPI887739 EPI887843 EPI887979 EPI917104 EPI918781 EPI918923 EPI919527 EPI926824 EPI926840 EPI971107 EPI971227 EPI971307 EPI979516 EPI979548

KC853231 KF259730 KJ195799 KJ946418 KP185531 KP413267 KP414050 KP415583 KP455980 KP657911 KP766136 KP766149 KP766150 KP766155 KP766156 KP766161 KP864449 KP864453 KU143379 KY221846 KY751112 KY751113 KY751131 KY751132 KY855520 KY855528 MF280192 MF370256 MF370264 MF629951 MF630031 MF630111 MF630199 MF630311 MF630327 MF630343 MF630447 MF630455 MF630471 MF630487 MF630511 MF630519 MF630535
